# The Efficacy of Encapsulated Phytase Based on Recombinant *Yarrowia lipolytica* on Quails’ Zootechnic Features and Phosphorus Assimilation

**DOI:** 10.3390/vetsci11020091

**Published:** 2024-02-15

**Authors:** Ekanerina A. Ovseychik, Olga I. Klein, Natalia N. Gessler, Yulia I. Deryabina, Valery S. Lukashenko, Elena P. Isakova

**Affiliations:** 1 Federal State Budget Scientific Institution Federal Scientific Center “Russian Research and Technological Poultry Institute” of Russian Academy of Sciences, Sergiev Posad 141311, Russia; ovseychik@vnitip.ru (E.A.O.); lukashenko@vnitip.ru (V.S.L.); 2 Bach Institute of Biochemistry, Research Center of Biotechnology of the Russian Academy of Sciences, Moscow 119071, Russia; klein_olga@list.ru (O.I.K.); gessler51@mail.ru (N.N.G.); yul_der@mail.ru (Y.I.D.)

**Keywords:** phytase, encapsulation, quail, feed additive, nutrient assimilation, *Yarrowia lipolytica*, *Obesumbacterium proteus*

## Abstract

**Simple Summary:**

In this study, we assayed a yeast-based phytase feed supplement for weight gain, slaughter yield, and assimilation of the feed using a domestic breed of quails. We have shown that the supplement containing heterologous bacterial phytase in the yeast cell impacted live weight more effectively than the addition of a commercial fungi phytase. The bacterial phytase in the yeast cells is supposed to be of higher activity in the gastrointestinal tract of the poultry and facilitates a more complete assimilation of plant products in the feed. The results obtained give a reason to consider that the application of intracellular phytase based on the yeast cells to the poultry feed improves quail growth performance and health status.

**Abstract:**

In this study, we used the Manchurian golden breed of quails. We assessed the efficacy of the food additives of the phytase from *Obesumbacterium proteus* encapsulated in the recombinant *Yarrowia lipolytica* yeast, which was supplied at a concentration of 500 phytase activity units per kg of the feed. One hundred fifty one-day-old quails were distributed into six treatment groups. The results showed that adding the *O. proteus* encapsulated phytase to the quails’ diets improved live weight, body weight gain, and feed conversion compared to those in the control groups and the groups using a commercial phytase from *Aspergillus ficuum*. The results obtained during the experiments indicate a high degree of assimilation of phytate-containing feeds if the encapsulated phytase was fed by the quails compared to that in the other groups. We can conclude that the class D encapsulated phytase is an expedient additive to the diets possessing better kinetic features compared to the PhyA and PhyC classes phytases when it acts inside the quail’s chyme.

## 1. Introduction

Currently, the world population needs an increasing amount of dietary meat, which promotes the dynamic development of a new branch of poultry farming, namely quail farming as a source of eggs and quail meat. Quail meat has a delicate consistency, juiciness, and aroma [1]. Quails show high egg productivity and rapid precocity. Hens start laying eggs at the age of 35–40 days, producing 250–280 eggs per year. Moreover, the feed expenses are 2.8–3.1 kg per 1 kg of eggs, and the weight of the eggs laid by a hen for a year exceeds the body weight of the hen 24-fold, whereas in the best chicken breeds, this ratio is 1:8 [2]. 

**Figure 1:**
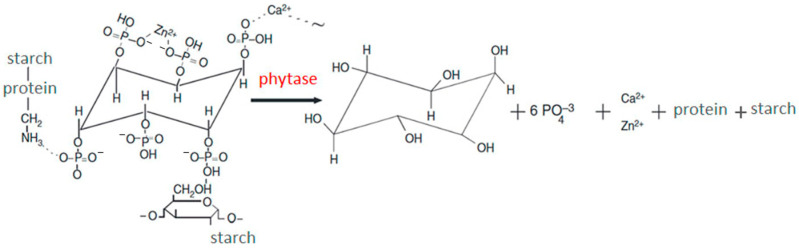
Scheme of phytate cleavage in the phytase reaction.

Successful, cost-effective breeding of the quails is only possible if the production technology can provide favorable conditions for poultry growth using the latest achievements in poultry husbandry technology [3,4,5,6]. For high productivity in the poultry industry, including quails, a balanced feed with phosphorus is an important component that should be applied. In the cells of all living organisms, there are two forms of phosphorus, namely ortho- and pyrophosphates. Moreover, phosphorus is included in phosphoproteins, phospholipids, nucleic acids, hexosе phosphates, etc. In vertebrates, phosphorus, along with calcium, is part of bone tissue. Myo-inositol hexakisphosphoric acid or phytic acid, being a myo-inositol derivative, i.e., a cyclic alcohol phosphorylated by six hydroxyl groups, is the main source of organic phosphorus in the plant feed for poultry farming (Figure 1) [7,8]. 

Both phytate phosphorus and mio-inositol only become available for assimilation upon being eliminated by phytase action. The phytase catalyzes the phytate hydrolysis (myo-inositol 1,2,3,4,5,6-hexakisphosphate) (Figure 1), which is the main phosphorus depot in plants and cannot be assimilated by poultry and pigs because of the insufficient phytase production in the gastrointestinal tract [9]. The grains of cereals, legumes, and seeds from oilseeds forming the basis of feed make up 60–88% of the total phosphorus. Since there are no phytases in the digestive tract in monogastric animals, they cannot assimilate most of the phosphorus from the plant feeds. In turn, the lack of phosphorus leads to evident disorders in the formation of animal skeletons. The use of feed phytase supplements in poultry and pig farming reduces the specific feed consumption spent per unit of production, which decreases the wastes generated and thereby increases the profitableness of agricultural production.

The current market offers a wide range of phytase preparations mainly based on the phytases of the microbial origins of class A (PhyA) and class C (PhyС) [10,11]. Actually, the phytases from *Escherichia coli* belonging to the PhyC class are the main type of feed phytases used in poultry farming. However, phytases of *E. coli* have a higher catalytic activity in the duodenal chyme, which allows them to obtain higher weight gain with less feed conversion. The microbial phytase intolerance to acidic pH (~1) in the poultry chymus is an obvious disadvantage of the actual phytase technologies. Moreover, the pH optimum for the activity of the PhyA (~2.2 and ~5.2) and PhyC (~5.5) phytase classes matches no pH in the poultry chymus where phytate hydrolysis occurs. The pH of the duodenum is 6.4–6.6, in the intestine it is ~6.5–7.0, and in the colon it is about 6.5–7.5 [12]. Thus, the excreta residual phosphorus upon using these phytases makes up 40 to 60% of the initial feed level. However, the phytase efficacy significantly increases if the phytases with a pH optimum of 6.0–7.5 are applied or if the phytases encapsulated in yeast cell micro-containers, which are tolerant in the poultry stomach and degrade in the chymus, are applied. 

However, while passing along the chyme of the poultry, the amount of enzyme significantly decreases due to acid denaturation and proteolysis. The technology of phytase encapsulation in yeast-producing cells as micro-containers can essentially decrease the loss of the enzyme and facilitate its thermal stability. Extremophilic *Yarrowia lipolytica* yeast is a promising object for modern biotechnology. In particular, yeast is used for the production of encapsulated phytase because of its annotated genome and its ability to create highly effective producers of recombinant proteins [12,13,14,15]. In our previous studies [16], we assayed the activity of the phytase in the transformant-producing intracellular phytase from *Obesumbacterium proteus* (OPP) encapsulated in the *Y.* lipolytica Po1f pUV3-Op yeast using the pUVLT2 vector based on the mitochondrial porin promoter of a voltage-dependent anion channel. The results obtained during the cultivation of the transformant pUV3-Op-2 in the fermenter confirmed the high activity of intracellular phytase upon the assimilation of phytate-containing substrates. The high stability of the encapsulated phytase of the Y. lipolytica pUV3-Oр transformant for heat treatment in a spray dryer was also noted [16,17]. In addition, we have received some encouraging results from the trial of the phytase supplement based on the transformant yeast using broiler chickens [18].

This study aims to assess the efficacy of the new encapsulated phytase additives based on the recombinant producer of Y. lipolytica Po1f pUV3-Op in poultry diets on quail productivity and phosphorus assimilability.

## 2. Methods and Materials

### 2.1. Experimental Description and Quail Husbandry

**Figure 2:**
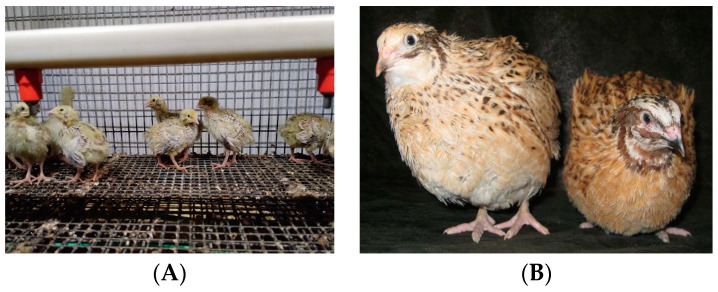
The quails of the Manchurian golden breed. (**A**) chicks; (**B**) the adult birds.

The research was performed in the Russian Research and Technological Poultry Institute of the Russian Academy of Sciences using the Manchurian golden quail (*Coturnix japonica*) (Figure 2) breed listed in the International Register of Quail Breeds and Lines. In total, 150 one-day-old Manchurian golden quails were placed in the battery cages (25 starters and growers in two cages for 42 days, and then they were divided into mixed-sex (roosters: hens, 1:4) groups (20/1394 cm^2^)). The birds were kept at a temperature of 30 °C for the first week, then it was reduced step by step to 22–23 °C by the fourth week. The humidity was maintained at 65 ± 5% during the whole experiment using a 12 h change of illumination (the light length was 580 nm, yellow). The quails had excess water and feed with one bird bath per nine quails. The quail sex was not distinguished until the age of 42 days. 

In the experiments, the roosters and hens were raised in the common cells as starters and then grew to their sexual maturity by the age of 42 days (Stage 1). Forty-two-day-old quails were separated into roosters and hens and transferred to the cage batteries for adult birds, where they were kept until 60 days of age with a sex ratio of 1:4 (roosters: hens) (Stage 2). Temperature and humidity, as well as the illumination in the room where the quails were kept, were measured using a combined device of the “TKA-PKM” series of a light intensity meter + thermo-hygrometer (Saint Petersburg, Russia).

The procedure of the experiments was accepted at the Local Ethics Committee meeting of The Federal Research Center “Fundamental Bases of Biotechnology” of Russian Academy of Sciences (Protocol No. 22/1, dated 7 September 2022). The birds were sacrificed using the committee-authorized method of animal care and scarification according to the European Union directive from 22 September 2010 (approval code: 2010/63/EC. The quails had excess water and feed with one bird bath per nine quails. The quail sex was not distinguished until the age of 42 days. 

Feeds were prepared from raw vegetable materials (corn, wheat, soybean meal, full-fat soybeans, sunflower meal, and vitamin premix with the addition of synthetic feed amino acids of lysine, methionine, and threonine but without raw materials of animal origin). For the first 28 days, the quails were fed using a starter diet (Table 1). The main feed used was ground; in the experimental groups, the supplements were applied in powdered form. Then, each bird was weighed, and the groups of 25 young quails each were formed with the analogous pairs technique. The experiment lasted for 60 days. Every group had unlimited access to feed and water. The feed was applied every day in the morning and evening excessively to monitor its consumption, and every portion and the remaining feed was weighed. The weight of the remaining feed was subtracted from the initial feed amount to calculate the consumed feed and FCR.

### 2.2. Dietary Plan

In the experiments, the pilot batches of feed additive containing encapsulated OPP and commercial Ladozym proxy phytase from *Aspergillus ficuum* (“BioPreparat”, Russia), with an activity of 5000 FYT/g, were used to study the efficiency of feeds with a total phosphorus of ~0.6% (available phosphorus 0.31–0.33%). The feed included (1) the first diet of food for quails from the first day of life to 4 weeks of age, (2) the second diet of feed for the quails of 5 to 6 weeks of age, and (3) the third diet for adult quails of the initial egg-laying period of 42 to 60 days of life. The feed was prepared using raw vegetable materials (corn, wheat, soy meal, full-fat soy, sunflower cake, and vitamin premix with some synthetic feed amino acids of lysine, methionine, and threonine). No raw animal materials were used. The total phosphorus in the three types of feed was 0.6 g per kg of feed, while the available phosphorus reached 0.35–0.45% g per kg of feed, which was 20–25% lower than that recommended for the quails. The feed for the three periods is shown in Table 1, Table 2 and Table 3.

In the experiment, six groups of one-day-old quails were formed, with 25 heads in each group, which differed in the types of feed:

*Control group 1* received the feed with total phosphorus of 0.8% (0.45% of available phosphorus) without any additives.

*Control group 2* received the feed with a total phosphorus of 0.6% (0.35% of available phosphorus) without any additives.

*Experimental group 3* received the feed containing total phosphorus of 0.6% (0.35% of available phosphorus) supplied with the encapsulated OPP at a dose of 500 FYT per kg of feed intake.

*Experimental group 4* received the feed containing total phosphorus content of 0.8% (0.45% of available phosphorus) supplied with the encapsulated OPP at a dose of 500 FYT per kg of feed intake.

*Experimental group 5* received the feed containing a total phosphorus content of 0.8% (0.45% of available phosphorus) supplied with the commercial phytase Ladozym proxy from *A. ficuum* at a dose of 4500 FYT per kg of feed intake.

*Experimental group 6* received the feed containing a total phosphorus content of 0.6% (0.35% of available phosphorus) supplied with the commercial phytase Ladozym proxy from *A. ficuum* at a dose of 4500 FYT per kg of feed intake.

### 2.3. The Phytase Activity Assay

To assay the activity of the phytase in the supplements, we used a colorimetric method assessing the phosphate ion released from the phytate, as described before [17]. A ten-milligram sample of each feed additive powder was placed in an Eppendorf tube. Then, 50 mg of glass beads (d = 0.3 mm) and 500 μL of 250 mM sodium acetate buffer (pH 5.5) were added to each tube and incubated in an ice-cold bath for 10 min. The swollen cells were homogenized by stirring with a hand vortex three times for 2 min each. The homogenates were clarified using centrifugation in a bench-top centrifuge at 14,000×*g* for 10 min [17]. The plate with the homogenates was incubated for 10 min at room temperature, and the absorbance was measured at 415 nm using a Uniplan plate spectrophotometer (Pikon, Saint Petersburg, Russia). The phytase activity unit (FYT) is calculated as amount of the enzyme releasing 1 µmol of the phosphate ion per minute under the above-mentioned conditions.

### 2.4. Feed Supplement Preparation Using the Yeast Strain Cultivation

Bio-supplements were prepared as described in [18] with some modifications. The *Y. lipolytica* yeast was grown in batches of 100 mL for 48 h at 28 °C using a full agarized medium with the composition (g/L): Bacto agar—20, yeast extract—10, Bacto Peptone—20, and glucose—20. The biomass was centrifuged, washed out, and used as an inoculum. For growth of the main culture, the following medium (g/L) was used: (NH_4_)_2_SO_4—_0.3; K_2_HPO_4—_0.5; Mg_2_SO_4—_0.5; KH_2_PO_4—_2.0; NaCl—0.1; and CaCl_2—_0.05, with different substrates. The wastes for substrate of fat of the chicken intestine were prepared as was described by Danilova et al. [18].

The cultural medium for the fermenter contained the following (g/L): yeast extract—10; broth from the chicken tripe—5; Bacto Peptone—20; and sophexyl antifoam—0.35 mL/L, with pH of 6.1. Then, a 3-liter fermenter with the medium was sterilized for an hour with a Miniforce Infors AG CH-4103 Bottmingen («INFORS HT», Bottmingen, Switzerland) equipped with controlled рН и рО_2_. The temperature was maintained at 28 °C, and the cultivation lasted for 21 h. The fermentation was performed at an aeration of 3 L/min and a rotation of 350 rpm.

**Figure 3:**
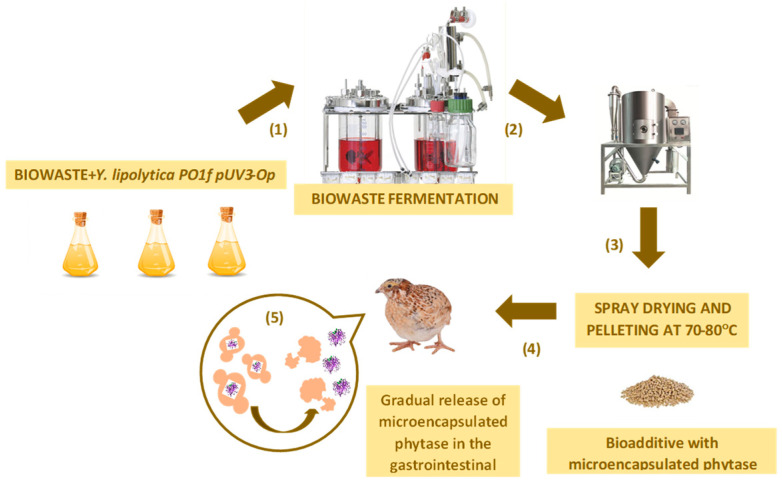
Scheme of the experiment for testing bioadditives on experimental animals [18]. (1) Production of the yeast biomass using the fermentation of the waste as the substrate (temperature—28 °C; rotation of 350 rpm; pH 5.5, at pH of 5.5 and 8.0). (2) Drying the product using a spray dryer (Mini Spray Dryer B–290, BÜCHI, Flawil, Switzerland) at 70 °C. (3) Grinding and granulation of the biomass. (4) The preparation of feed mixtures based on the recombinant *Y. lipolytica PO1f* (*pUV3-Op*) transformant. (5) The analysis of the efficacy of the encapsulated phytase in the *Y. lipolytica PO1f* (*pUV3-Op*) transformant for increasing the phosphorus bioavailability and reducing anti-nutritional phytate activity of plant feed using the model of quails compared to the efficacy of the commercial phytase.

The pH and oxygen pressure of the fermentation medium were monitored using a polarographic oxygen sensor. The main fermentation was performed in a fermenter with a cultivation volume of 7.5 L (LabMakelaa, Baar, Switzerland). The medium was of the following composition (g/L): Bacto Peptone (Difco, Leeuwarden, The Netherlands)—20; yeast extract (Difco, Leeuwarden, The Netherlands)—10; and the broth from chicken tripe—5. The broth from chicken tripe was sterilized at 120 °C for 40 min. The cultivation lasted for 72 h at 28 °C under a rotation of 350 rpm with sterile air (pH of 5.5). A total of 150 mL sophexyl in 500 mL distilled water was used as antifoam solution and sterilized for 40 min at 120 °C. Cultivation pH was controlled, and the oxygen concentration was monitored with the polarographical electrode with a Teflon membrane (Sea&Sun Technology, Germany, Trappenkamp) (Figure 3).

The cells were concentrated from the fermentation medium with flow separation and were dried in a spray drying at 75 °C (Buchi Mini Spray Dryer B-290, Flawil, Switzerland). The obtained powder was weighed, and the phytase activity was assessed (Figure 3).

### 2.5. Sample Collection and Processing

To determine the quality of the meat and internal organs of the bird, the slaughter of some 42-day-old quails was performed. Three hen quails and three cock quails were selected from each group. Upon an anatomical dressing of the poultry, the average weight of the carcasses, the slaughter yield, and the yield of the main parts of the carcasses, including the main internal organs, were assayed.

### 2.6. The Assay of The Experimental Feeds and the Quail Excreta Composition

To perform the element assessment, we dried the samples at 60 °C until reaching the stable weight, 10 g aliquots were crushed, and the aliquot of 0.5 g was assayed. The feed chemical compositions used in the groups and the quail excreta were assessed at Dokuchaev Soil Science Institute, RAS. They used the energy-dispersive X-ray fluorescence EDXRF analysis (Thermo Scientific, Waltham, MA, USA) as was described before in Tabinda and Butt [19].

### 2.7. Feed Conversion Ratio (FCR)

The FCR was calculated as described by Ziarat et al. [20].

### 2.8. Assay of Phosphorus and Calcium Level in the Tibiae of the Quails

To assess the bone characteristics, the tibiae of the quail’s right leg were removed, stripped, cleaned, defatted, and submerged in ethyl ether for 72 h. Then, it was dried at 100 °C and weighed precisely. After weighing, it was calcined at 600 °C in a muffle furnace for 12 h. Afterward, it was ground to assay the calcium and phosphorus amount as described by Shastak et al. in [21].

### 2.9. Statistics

To calculate the standard error of the mean (SEM), the data obtained were treated using STATISTICA 10 (StatSoft, Inc., Tulsa, OK, USA, www.statsoft.com, accessed on 15 September 2022) as was described by Norton et al. in [22]. We analyzed the data as group mean values ± standard deviation (mean ± SD) and compared the different groups using ANOVA. Differences are considered statistically significant at *p* < 0.05. The project involved six groups of animals, with 25 quails in each. The pool of 10 animals was considered an experimental unit for the variables: body live weight, weight gain, carcass yield, and cut yields. All statistical analyses were realized using R language, version 4.3.1 (Free Software Foundation, Boston, MA, USA, https://www.r-project.org/, accessed on 15 October 2023). Quail body weight differences throughout the 6-week experiment were examined for normal distribution using Shapiro–Wilks tests. Then, the tests for the homogeneity of covariance matrices using the box_m function and tests for equality of variances using Bartlett’s test function [23] were performed. We analyzed the data as group mean values ± standard deviation (mean ± SD) and compared the different groups using one-way ANOVA. After that, Duncan multiple range tests were used to compare the means of all the groups (control group 1, control group 2, group 3 (0.35% of available P + encapsulated OPP (500 FYT per kg of feed), group 4 (0.45% of available P + encapsulated OPP (500 FYT per kg of feed)), and group 5 (0.45% of available P + Ladozym proxy from *A. ficuum* (4500 FYT per kg of feed)). *p* < 0.05 was used to declare significance. The tests showed significant differences between the means of groups 1 and 4 and groups 4 and 5 for quail weight differences at 5 and 6 weeks.

## 3. Results

This study was performed in two stages. All the animals were caged together (Stage 1) for 42 days. On the 42nd day, the animals were separated by sex into hens and roosters in a ratio of 1:4, and we assessed all the indicators up to 60 days (Stage 2). Figure 3 shows the scheme of the tests performed.

### 3.1. Stage 1

#### 3.1.1. Growth Performance at the Age of 42 Days

In the first stage of this study, the productivity, feed assimilability, and FCR of the 42-day-old quails were assayed. Table 4 shows the results of the quails’ live weight from 1 day to 42 days of age. The daily body weight of quails in all the studied groups was equal, being, on average, 8 g. The difference in the body weights of the quails from 7 to 28 days of age in all the groups tested differed insignificantly (Table 4). However, at the age of 28 days, the body weight in experimental group 4 using the encapsulated OPP at a dose of 500 FYT per kg of feed and the total phosphorus content of 0.8% (0.45% of available phosphorus) exceeded that in control group 1 by 1.42% and exceeded that in control group 2 by 1.87% (Table 4).

**Figure 4:**
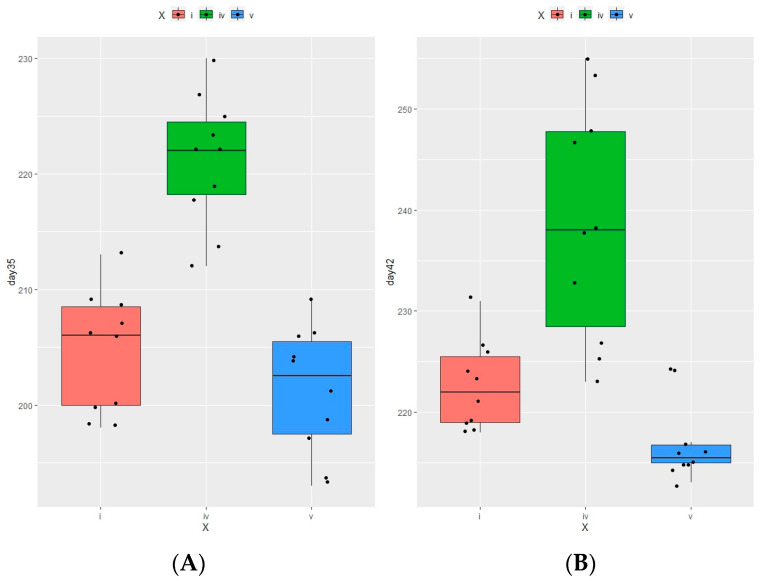
Calculated correlations (*p*-values, function ‘cor_test’) with a significance level ≤ 5% between body weight variations in the samples of the same type. *p*-values were obtained for all the pairs of live weights (for different weeks) (Figures S1 and S2). (**A**)—on day 35; (**B**)—on day 42. Footnotes: i—group 1 (0.45% of available P without any additives); iv—group 4 (0.45% of available P + encapsulated *OPP* (500 FYT per kg of feed); v—group 5 (0.45% of available P + Ladozym proxy from *A. ficuum* (4500 FYT per kg of feed). *n* = 10 in each group.

By the 35th day of life, the average body weight in group 4 was higher than that in the positive control (group 1) by 2.13% (Figure 4), whereas in groups 5 and 6, it decreased by 0.36% and 2.44%, respectively (Table 4, Figure 4). Table 4 and Figure 4 show that the highest body weight of 240.4 g among the hens was noted in experimental group 4, whereas in both control groups, it was 218.5 g for the first one and 216 g for the second one, respectively. The live weights of the hens in experimental groups 3 and 4 compared to those in experimental group 6 were higher by 1.3 and 10.46, respectively (Table 4).

As for the live weight of the roosters, experimental group 4 showed the highest gains. The average body weight of the roosters in experimental group 4 was 218.1 g, which was higher by 1.1 and 3.2% than that in control groups 1 and 2, respectively (Table 4). In experimental group 4, it was higher by 4.0 and 8.4% compared to that in experimental groups 5 and 6, respectively. It is worth noting that the data for experimental group 4 were statistically significantly different (at *p* < 0.05) from those in control group 1 and experimental group 5 (Table 4, Figures S1 and S2). Hence, the total average live weight of the quail in experimental group 4 was higher by 0.2 and 1.2% than those in control groups 1 and 2, respectively. And it was higher by 3.3 and 5.5% compared to those in experimental groups 5 and 6, respectively (Table 4).

The results of the quail growth up to 42 days were analyzed, and the main zootechnic productivity indicators were calculated (Table 5). The data in the table display that during the 42-day experiment, the percent alive in all the studied groups reached 100%; the highest average daily gain of the poultry during the whole growing period was observed in experimental group 4, where it amounted to 5.00 g (Table 5). The lowest average daily gains were found in experimental groups 5 and 6, with those being 3.87 and 3.89 g, respectively.

Feed conversion is the most important zootechnic feature describing how many units of feed were spent per unit of live weight gain. The lowest feed expense per 1 kg of live weight gain was obtained in experimental group 4. Thus, in experimental group 4, the feed expenses were lower by 0.5 and 1.8% compared to those in control groups 1 and 2 and by 3.6 and 4.1% compared to those in experimental groups 5 and 6 (Table 5). The FCR in groups 3 and 4 decreased by 1% and 2% compared to that in control groups 1 and 2, respectively (Table 5). However, FCR in groups 5 and 6 using the commercial phytase increased by more than 2% (Table 5).

#### 3.1.2. Carcass Criteria and Organs on the 42nd Day of Life

**Figure 5:**
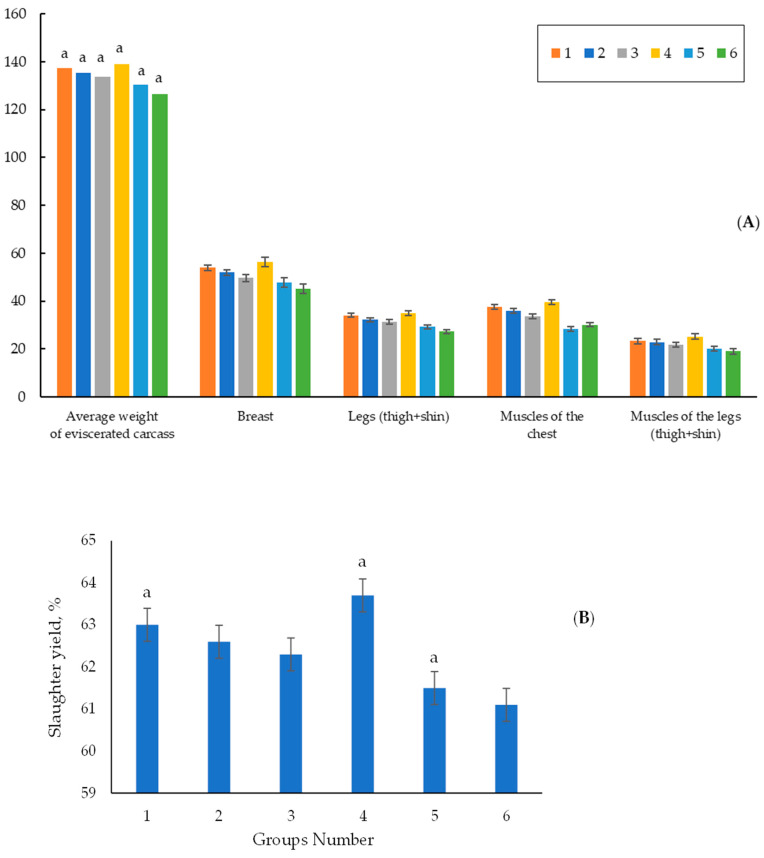
Meat qualities (**A**) and slaughter yields (**B**) of 42-day-old quails. Footnotes: Groups: 1—0.45% of available P without any additives; 2—0.35% of available P without any additives; 3—0.35% of available P + encapsulated *OPP* (500 FYT per kg of feed); 4—0.45% of available P + encapsulated *OPP* (500 FYT per kg of feed); 5—0.45% of available P + Ladozym proxy from *A. ficuum* (4500 FYT per kg of feed); 6—0.35% of available P + Ladozym proxy from *A. ficuum* (4500 FYT per kg of feed). a—the difference in the weight of the gutted carcass in the experimental groups at *p* ≤ 0.05 (Figure S3). Unlabeled data were not statistically different. *n* = 6 in each group.

To assay the quality of the meat and the internal organs of the poultry, the quails were killed at the age of 42 days. Figure 5 shows the data on the eviscerated carcass weight (on average for roosters and hens), slaughter yield, the mass of the main parts, and the muscles. Experimental group 4 had the highest average weight of the eviscerated carcass, with this being 139.0 g.

**Figure 6:**
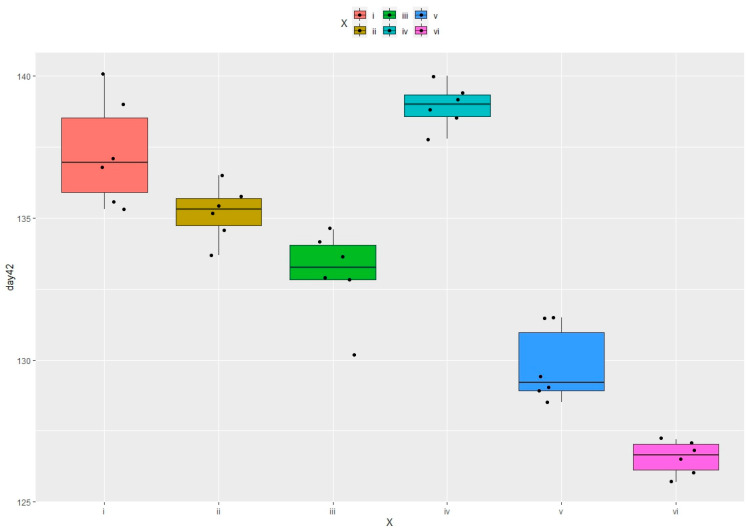
Calculated correlations (*p*-values, function ‘cor_test’) with a significance level ≤ 5% between carcass weight variations in the samples of the same type. *p*-values were obtained for all the pairs of carcass weights on the 42nd day (Figure S3). Footnotes: i—group 1 (0.45% of available P without any additives); ii—0.35% of available P without any additives; iii—0.35% of available P + encapsulated OPP (500 FYT per kg of feed); iv—group 4 (0.45% of available P + encapsulated *OPP* (500 FYT per kg of feed); v—group 5 (0.45% of available P + Ladozym proxy from *A. ficuum* (4500 FYT per kg of feed); vi—0.35% of available P + Ladozym proxy from *A. ficuum* (4500 FYT per kg of feed). *n* = 6 in each group.

The weight of the eviscerated carcasses in experimental group 4 was higher by 2.6 and 2.8% than those in control groups 1 and 2, respectively. In experimental group 4, it was higher by 6.6% (at *p* < 0.05) and by 9.9% (at *p* < 0.05) than those in experimental groups 5 and 6 (Figure 6 and Figure S3). In experimental group 3, the average weight of the eviscerated carcass was slightly lower than those in control groups 1 and 2 and in experimental group 4 (Figure 5A). The best data for slaughter yield based on the weight and fatness of carcasses were obtained in experimental group 4. It was higher by 0.7 and 1.1% compared to those in control groups 1 and 2 and was higher by 2.2 and 2.6% compared to those in experimental groups 5 and 6. The slaughter yields in experimental group 3 were higher by 0.8 and 1.2% than those in experimental groups 5 and 6, respectively (Figure 5B).

The results of the anatomical cutting of the main parts and muscles of the carcasses (breast and ham) are presented in Table 6.

Experimental group 4 showed the highest breast yield, with this being 40.5 g (Table 6). It was higher by 1.2 and 2.1% than those in control groups 1 and 2, and it was higher by 3.9 and 4.8% compared to those in experimental groups 5 and 6, respectively. 

The yield of the pectoral muscles in experimental group 4 was higher by 1.1 and 1.9% and 4.2 and 4.6% compared to those in control groups 1 and 2 and in experimental groups 5 and 6, respectively (Table 6). Muscle yield in the legs in experimental group 4 was higher by 1.1 and 1.2% and by 2.6 and 3.0% compared to those in control groups 1 and 2 and experimental groups 5 and 6, respectively. In experimental group 3, the output of pectoral muscles and muscles in the legs was higher by 0.7 and 1.1% and 1.1 and 1.5% than in experimental groups 5 and 6, respectively (Table 6).

**Figure 7:**
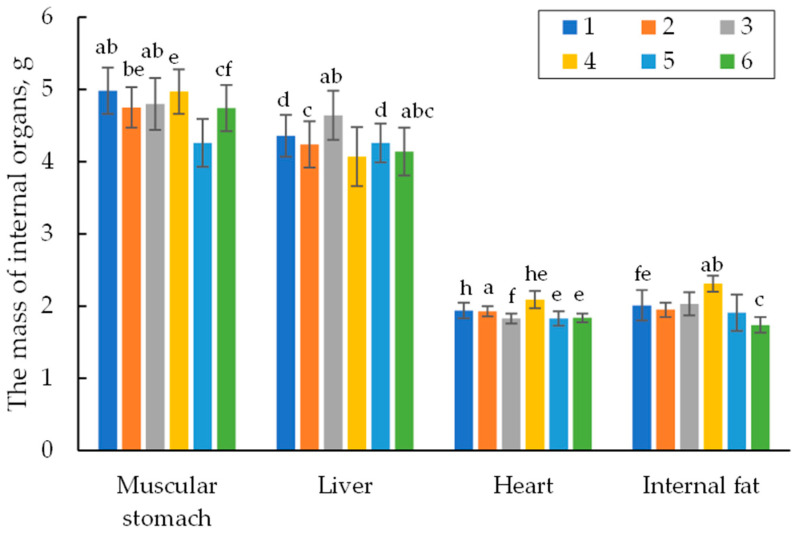
The weight of the internal organs of quails on the 42nd day. Footnotes: Groups: 1—0.45% of available P without any additives; 2—0.35% of available P without any additives; 3—0.35% of available P + encapsulated *OPP* (500 FYT per kg of feed); 4—0.45% of available P + encapsulated *OPP* (500 FYT per kg of feed); 5—0.45% of available P + Ladozym proxy from *A. ficuum* (4500 FYT per kg of feed); 6—0.35% of available P + Ladozym proxy from *A. ficuum* (4500 FYT per kg of feed). Mean values are displayed (*n* = 3, ±SD): a—*p* < 0.04; b—*p* < 0.01; c—*p* < 0.001; d, e—*p* < 0.02; f—*p* < 0.002. *n* = 6 in each group.

Upon cutting, the internal organs of the quails in each group were weighed and assayed. Figure 7 shows the data on the weight of the main internal organs of the 42-day-old quails. The internal organs, namely the gizzard, liver, and heart, were within the physiological standard. Moreover, there was no significant difference in the weight of the main internal organs among the groups studied. The amount of abdominal fat in all the groups tested was also almost at the same level.

#### 3.1.3. Chemical Composition of Quails’ Bones

Table 7 shows the mineral residue (%) in the tibiae bones of the quails. The phosphorus amount in the quails’ bones in group 4 was 6.68% higher than that in control group 1, and it was higher by 0.8% and 1.3% compared to those in groups 5 and 6. Moreover, the calcium level increased in groups 4–6 by approximately 8% compared to that in control group 1. It indicates the impact of the microencapsulated OPP applied to the feed on the phosphorus balance in the tibia bones of the quails.

### 3.2. Stage 2

#### 3.2.1. Growth Performance of the Quails at the Age of 60 Days

In the second stage of the experiment, the productivity, feed assimilability, and FCR of the adult quails were studied for the period from 42 to 60 days of age. So, the 42-day-old quails of similar body weight were transferred to the cell batteries with a sex ratio of roosters and hens of 1:4. The adult quails were weighed at the age of 60 days, and the average live weights of hens and roosters were calculated for each group. Moreover, their total average live weight was calculated, too. The results are presented in Table 8.

Both experimental group 4 and control group 1 showed the highest average body weight of 264.7 g in experimental group 4 and 260.9 in control group 1 (Table 8). The live weight of hens in experimental group 4 exceeded those in experimental groups 5 and 6 by 7.2 and 10.9%, and the live weight of the roosters exceeded them by 11.1 and 8.9%, respectively (Table 8). It was statistically significantly higher in experimental group 4 compared to the live weight of the hens in both control group 2 (at *p* ≤ 0.05) and experimental group 6 (at *p* ≤ 0.05). Moreover, that of the roosters in experimental group 4 was significantly higher compared to that in control group 2 (at *p* < 0.05) and in both experimental groups 5 and 6 (at *p* < 0.05) (Table 8).

Table 9 shows the main zootechnic features of the adult quails from 42 to 60 days of life. The highest average daily gain of the poultry during the whole growing period was also observed in experimental group 4, whereas the feed consumption per single average live weight was the lowest in both experimental groups 3 and 4. In experimental group 4, they were lower by 3.5 and 9.0% compared to those in control groups 1 and 2 and 8.2 and 9.3% lower than those in experimental groups 5 and 6, respectively (Table 9). However, the similar indicators in groups 5 and 6 using the commercial phytase were either comparable to the control ones or even slightly higher than they were.

#### 3.2.2. Carcass Criteria, Organs, and Egg Laying Productivity on the 60th Day of Life

The intensity of egg production is the key feature of productive poultry. It is determined by the ratio of the number of laid eggs for a certain time to the number of laying hens, expressed as a percentage. According to the calculation results, the highest intensity of egg production, 86.8%, was detected in experimental group 4. Thus, the intensity in experimental group 4 was 9.7 and 13.2% higher than those in control groups 1 and 2, and it was 11.8 and 9.0% higher than those in experimental groups 5 and 6, respectively (Table 9).

**Figure 8:**
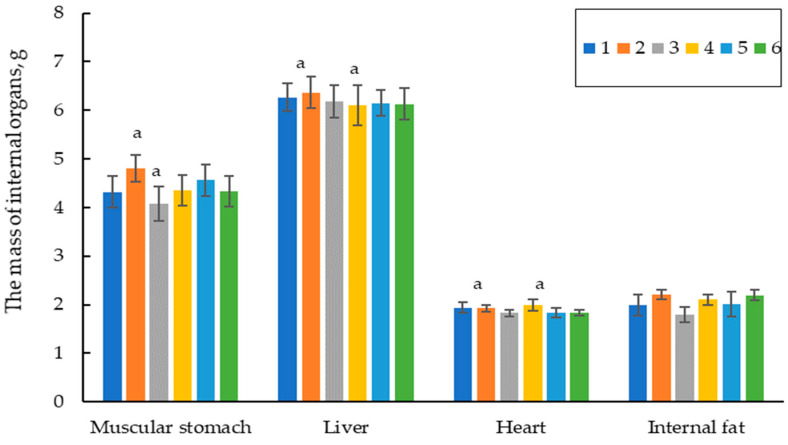
The weight of internal organs of 60-day-old quails. Footnotes: Groups: 1—0.45% of available P without any additives; 2—0.35% of available P without any additives; 3—0.35% of available P + encapsulated *OPP* (500 FYT per kg of feed); 4—0.45% of available P + encapsulated *OPP* (500 FYT per kg of feed); 5—0.45% of available P + Ladozym proxy from *A. ficuum* (4500 FYT per kg of feed); 6—0.35% of available P + Ladozym proxy from *A. ficuum* (4500 FYT per kg of feed). a—the difference in the weight of the gutted carcass in the experimental groups at *p* ≤ 0.05. Unlabeled data were not statistically different. *n* = 10 in each group.

Figure 8 shows the results of the quails’ eviscerated carcasses (on average) for roosters and hens. Experimental group 4 showed the highest average weight of the eviscerated carcass of 165.9 g, which was 1.7 and 11.5% higher than those in control groups 1 and 2, respectively. The advantage of experimental group 4 concerning liver, heart, and gizzard compared to those in control group 2 was highly statistically significant at *p* ≤ 0.05 (Figure 8). 

#### 3.2.3. The Effect of the Phytases on Residual Phosphorus and Macro- and Microelements in the Quails’ Excreta

The balance experiments were performed using the 14-day-old and 28-day-old quails (Table 10 and Table 11). Ten quails were selected from each group in separate cages. The litter samples were collected from each group for three days. Those litter samples were assayed for macro- and microelement composition. The assessment of the experimental feed composition and quails’ excreta for phosphorus amount showed that by the 28th day of the experiment, the level of residual phosphorus in groups 3 and 4 (feed supplemented with encapsulated phytase at a dose of 500 FYT per kg of feed) decreased by 10% and 7% compared to that in control group 1. However, in group 5, using the commercial phytase Ladozym proxy from *A. ficuum* (PhyA) in an excess dose of 4500 FYT per kg of feed, the phosphorus level increased by 9.5% (Table 10 and Table 11). On the 42nd day of the experiment, the phosphorus level in the quails’ excreta of group 4 decreased by 0.8%, while in group 5, it increased by 7.7%.

The residual phosphorus in the feces nearly halved upon using the encapsulated phytase compared to those in both control groups. It could indicate the high efficacy of the encapsulated phytase in the chyme of the quails, unlike the extracellular enzyme. The application of the encapsulated enzyme at a high dose (500 FYT/kg of feed) significantly declined the amount of main macro- and microelements, namely Mg, K, Ca, Zn, and Cu (Table 11).

## 4. Discussion

Animals obtain phosphorus from plant feeds mainly as hardly soluble phytates. In monogastric animals, including poultry, the only sources of phytases for releasing phosphate groups from phytates are plant phytases coming from feed and their intestinal microflora. Phytases are enzymes of the phosphatase class that catalyze the cleavage of phytic acid to inorganic phosphate [24]. They are reported to strengthen bones and facilitate the efficiency of animal nutrition [24,25,26]. Phytase dietary supplements improve intestinal health by reducing the secretion of the gastrointestinal tract, which leads to increased productivity and energy use [27]. However, because of various reasons (low activity of phytases in feedstocks, a decrease in their activity during heat treatment and feed storage, the difference in the pH optimum of the enzyme, and the acidity of the gastrointestinal tract), the assimilation of phytates in the tract exceeds no more than 10%, and the remaining need for phosphorus should be substituted by mineral additives [28]. Most of the phytates from the animal feed are not digested and are excreted into the external environment with feces. Phytic acid binds cations, so its high level in the diet, for example, in grain-based feeds, could cause a deficiency of some elements, namely calcium, iron, zinc, etc., and reduce their assimilation. [29]. The main trend in solving the problem is to decrease the introduction of mineral additives and to apply phytases to provide the animals with phosphorus from phytates [30,31,32].

The microbial enzyme instability at acidic pH (~1) in the poultry stomach is an apparent disadvantage of the current phytase technologies. Moreover, no pH optimum of the classes PhyA phytases (~2.2 and ~5.2) and the PhyC class one (~5.5) matches the pH values in the poultry chymus where phytate is hydrolyzed: in the duodenal section, the pH is 6.4–6.6; in the intestine, it is 6.5–7.0; and in the colon, it is 6.5–7.5 [33]. It proves that the residual phosphorus in the excreta, even upon using the phytases, is 40 to 60% of its original level in the feed. This necessitates the application of either phytase with a pH optimum within 6.0–7.5 or the encapsulation of phytases in the micro-containers, which are tolerant in the stomach of the poultry, however, it can degrade in the chymus. It should significantly increase the efficiency of phytases.

Before, in our studies using the phytase from *O. proteus* (the Enterobacteriaceae family), a technology for microencapsulation of phytase inside the cells of the *Y. lipolytica* extremophilic yeast was proposed. The encapsulation had success if the secretory systems were blocked upon designing the producer. There was some drop in the target enzyme yield, on average, from 15,000–40,000 FYT/L in a secretory producer based on the same yeast species to 1000–1500 FYT/L. However, it significantly improved the technological features of the producer since the enzyme preparation does not concentrate and drying, which reduces the energy costs by 10–50 times and makes the technology completely waste-free [15]. An essential increase in the phytase stability in the stomach of the animal was the main result. It should be noted that the approach in this study supposes that the phytase encapsulated in the yeast, due to its high efficiency, can be added to the feed at rather low concentrations (not more than 1.5%) compared to that of the commercial phytase used as the control. In the experiments using mice, we observed significantly higher weight gain at a dose of encapsulated enzyme of 100 FYT per kg of feed than upon using the commercial water-soluble phytase from *A. ficuum* at a dose of 3000 FYT per kg of feed [34].

The data obtained in the experiments, including the results on the average live weight, the weight gain of the quails (Table 4 and Table 6), FCR (Table 5), slaughter yield, and pectoral muscle, thigh, and leg weight of the poultry (Figure 5 and Figure 7), indicate that the encapsulated phytase showed higher efficiency on the productivity of the quails than extracellular phytase from *A. ficuum*, belonging to the class of water-soluble phytases of the PhyA and PhyC. In the paper by [35], the authors studied the efficacy of the phytase-containing supplement of Agrofit in the feeds for the meat quails. They found that feeding phytase in the amount of 75 g/t of the feed (~330 FYT per kg of feed) increased the average daily gain in live weight of the quails by 4.8%, increased the percent alive by 1.5% and improved the feed conversion by 12.2%. The enzyme preparation of Agrofit (“Agroferment”, Russia) contains the enzyme phytase with an activity of at least 5000 units/g (the producer of *Penicillium canescens* PhPl-33 BKM F-3867D), and according to its features, it can belong to class A phytases. A comparative analysis of the productivity of the experimental groups used by the authors and the results obtained in our experiments showed that under similar feeding conditions (0.4–0.45% of available phosphorus, 500 FYT a kg of feed), we obtained a similar productivity of 1.2% versus 1.3% stated in the paper and comparable feed costs (3.73–3.71 kg per 1 kg of live weight gain). However, it should be noted that, unlike the composition of the feed used by the authors (fish flour, 7.98%, and meat and bone flour, 5.26%), the feed in our experiments contained no animal products, and the phytase preparation we applied kept 100% of the quail population alive throughout the experiment unlike that in the paper (97.5%). Thus, we have practically confirmed that the encapsulated phytase from *O. proteus* applied to the quail feed is highly effective even compared to the commercial preparations. 

It should be noted that there are some studies in the references [26,35] where various aspects of the phytase effect on the morphological and biochemical features of the quails and the properties of their eggs and meat were assayed. However, there are no comparative data on the effect of water-soluble phytases on the productivity of the quails, which makes it difficult to compare the data obtained in our experiments to the results of other authors. In the study by [36], it was shown that the introduction of 500–2500 FYT per kg of feed (Ronozyme P, Roche Corporate Headquarters, Basel, Switzerland), along with xylanase, released the transportation stress in Japanese quails (*Coturnix Coturnix japonica*). In the paper by [37], the positive role of phytase (Natuphos E 10000, BASF, Brazil) in the concentration of 300 FYT /kg of feed in daily weight gain of the quails fed without animal components that had a phosphorus deficiency of 65% was proved. The authors concluded that phytase itself suppresses the negative effects of moderate phosphorus restriction in the cultivation of Japanese quails but does not cope with it upon strict phosphorus restriction. In another paper, Japanese quails were fed with diets containing phytase at the dose of 500 and 1000 FYT per kg of the feed, and excellent results were obtained in FCR [38]. Moreover, in the paper by [35], using the quails, there were no positive effects of the phytase (Natuphos 500) at a dose of 1000 FYT per kg of the feed on poultry productivity. Ravindran and co-authors [39] supposed that the difference between the obtained effects using diets with a high phytase concentration could be due to some effectors, including the phytase origin, ingredients, and dietary features in each study using the poultry.

The results of the composition of the feed and the quail excreta of phosphorus level showed that on the 28th day of the experiment, the level of residual phosphorus in groups 3 and 4 (feed with the encapsulated *OPP* at a dose of 500 FYT a kg of feed) decreased compared to that in control group 1 by 10% and 7%, respectively. In group 5, using the commercial phytase Ladozym proxy from *A. ficuum* (PhyA) at an abundant dose of 4500 FYT a kg of feed, the phosphorus level even increased by 9.5% (Table 11). By the forty-second day of the experiment, the phosphorus level in the excreta of group 4 decreased by 0.8%, while in group 5, it increased by 7.7%. Similar data were obtained by us before in the experiments on broiler chickens [18]. Taken together, these data argue for a high level of phytate-containing assimilation in the presence of encapsulated phytase by the quails and broiler chickens compared to both the control and the commercial phytase preparation of the PhyA class.

In the second stage of the experiment, we first assayed the quail egg production and demonstrated that the highest one was obtained in experimental group 4, where it reached 86.8% (Table 9), exceeding those in 1, 2, 5, and 6 by 9.7, 13.2%, 11.8, and 9.0%, respectively (Table 9). The positive effects of the phytase on egg production and egg properties are well known for laying hens. So, Lim et al. [40] showed that the application of the microbial phytase at a dose of 300 FYT per kg of feed can improve egg production and reduce the percentage of broken and soft eggs and phosphorus excretion. Furthermore, the effects of phytase supplements depended much on the level of Ca and non-phytate phosphorus. The interaction between the level of the phytase and non-phytate phosphorus showed that phytase increased egg production in the chickens with a diet containing 0.25% phosphorus but not in the chickens using a diet with 0.15% non-phytate phosphorus and 4.0% Ca. Casartelli et al. [41] assayed the effect of phytase (0 and 100 FYT/kg) in diets including various sources of phosphorus and calcium (Ca and sodium phosphate, microgranulated di-Ca phosphate, and triple superphosphate). They showed that the application of the phytase significantly affected the egg production traits.

## 5. Conclusions

The data of the experiments first showed that a high degree of assimilation of phytate-containing feeds upon using the encapsulated phytase might be beneficial in the poultry industry. The quail chicks, which received the feed supplement of the encapsulated phytase based on the *Y. lipolytica* yeast, demonstrated good live weight indicators, improved meat quality, and assimilability of phosphorus and calcium. We can conclude that the supplement of class D encapsulated phytase to the diets could be rather expedient when it acts inside the quail chyme. More thorough research is still required to determine in what chyme section the main degradation of the phytate-containing feeds occurs.

## Figures and Tables

**Table 1:** The composition of the basal diet for the 1-day-old to 4-week-old quails (%) (period 1).

1 kg of the Feed Contains (%)	Groups					
	Group 1	Group 2	Group 3 (9 g/kg)	Group 4 (9 g/kg)	Group 5 (3 g/10 kg)	Group 6 (9 g/10 kg)
Soybean meal	42.54	42.42	42.91	43.06	42.40	42.46
Wheat	28.60	29.14	27.23	26.68	29.19	28.95
Corn	20.00	20.00	20.00	20.00	20.00	20.00
Sunflower oil	4.77	4.62	5.14	5.29	4.61	4.67
OPP	-	-	0.90	0.90	-	-
Ladozym proxy	-	-	-	-	0.03	0.09
Monocalcium phosphate	1.50	0.99	0.99	1.51	0.89	0.99
Limestone Ca	1.19	1.44	1.44	1.18	1.49	1.44
Premix	0.50	0.50	0.50	0.50	0.50	0.50
Salt	0.31	0.31	0.31	0.31	0.31	0.31
Methionine	0.27	0.26	0.27	0.27	0.26	0.27
Lysine	0.09	0.09	0.08	0.08	0.09	0.09
Threonine	0.08	0.08	0.08	0.08	0.08	0.08
Choline chloride	0.08	0.08	0.08	0.07	0.08	0.08
Soda	0.06	0.06	0.06	0.06	0.06	0.06
FEKORD enzyme	0.01	0.01	0.01	0.01	0.01	0.01
Total	100	100	100	100	100	100
100 g of the feed contains (%)						
Metabolizable energy (kcal)	300	300	300	300	300	300
Crude fiber	24.85	24.85	24.85	24.85	24.85	24.85
Raw fiber	4.28	4.28	4.27	4.26	4.28	4.28
Calcium	1.00	1.00	1.00	1.00	1.00	1.00
Total phosphorus	0.77	0.65	0.65	0.77	0.63	0.65
Phosphorus assimilated	0.45	0.35	0.35	0.45	0.45	0.35
Sodium	0.16	0.16	0.16	0.16	0.16	0.16
Chlorine	0.23	0.23	0.23	0.23	0.23	0.23
Lysine assimilated	1.23	1.23	1.23	1.23	1.23	1.23
Methionine assimilated	0.58	0.58	0.58	0.58	0.58	0.58
Threonine assimilated	0.85	0.85	0.85	0.85	0.85	0.85

Footnotes: 1—0.45% of available P without any additives; 2—0.35% of available P without any additives; 3—0.35% of available P + encapsulated OPP (500 FYT per kg of feed); 4—0.45% of available P + encapsulated OPP (500 FYT per kg of feed); 5—0.45% of available P + Ladozym proxy from A. ficuum (4500 FYT per kg of feed); 6—0.35% of available P + Ladozym proxy from A. ficuum (4500 FYT per kg of feed). FEKORD is a nutritional fodder ferment additive; OPP is intracellular phytase from Obesumbacterium proteus encapsulated in the Y. lipolytica Po1f pUV3-Op yeast.

**Table 2:** The composition of the basal diet for the 5–6-week-old quails (%) (period 2).

1 kg of the Feed Contains (%)	Groups					
	Group 1	Group 2	Group 3 (5 g/kg)	Group 4 (5 g/kg)	Group 5 (3 g/10 kg)	Group 6 (9 g/10 kg)
Soybean meal	11.11	11.03	10.10	10.18	11.12	11.06
Wheat	54.79	55.14	54.64	54.30	54.75	55.02
Corn	15.00	15.00	15.00	15.00	15.00	15.00
Sunflower seed cake	13.39	13.37	13.82	13.84	13.39	13.37
Sunflower oil	1.00	1.00	1.00	1.00	1.00	1.00
OPP	-	-	0.50	0.50	-	-
Ladozym proxy	-	-	-	-	0.03	0.09
Monocalcium phosphate	1.56	1.05	1.06	1.57	1.56	1.05
Limestone Ca	1.87	2.13	2.13	1.87	1.87	2.13
Premix	0.50	0.50	0.50	0.50	0.50	0.50
Salt	0.36	0.36	0.36	0.36	0.36	0.36
Methionine	0.03	0.03	0.04	0.04	0.03	0.03
Lysine	0.30	0.30	0.37	0.36	0.30	0.30
Threonine	-	-	0.39	0.39	-	-
Choline chloride	0.08	0.08	0.08	0.08	0.08	0.08
The FEKORD enzyme	0.01	0.01	0.01	0.01	0.01	0.01
Total	100	100	100	100	100	100
100 g of the feed contains (%)						
Metabolizable energy (kcal)	2750	2750	2750	2750	2750	2750
Crude fiber	17.00	17.00	17.00	17.00	17.00	17.00
Raw fiber	5.00	5.00	5.00	5.00	5.00	5.00
Calcium	1.20	1.20	1.20	1.20	1.20	1.20
Total phosphorus	0.80	0.68	0.68	0.80	0.80	0.68
Phosphorus assimilated	0.45	0.35	0.35	0.45	0.45	0.35
Sodium	0.16	0.16	0.16	0.16	0.16	0.16
Chlorine	0.27	0.27	0.27	0.27	0.27	0.27
Lysine assimilated	0.76	0.76	0.76	0.76	0.76	0.76
Methionine assimilated	0.29	0.29	0.30	0.30	0.29	0.29

Footnotes: 1—0.45% of available P without any additives; 2—0.35% of available P without any additives; 3—0.35% of available P + encapsulated OPP (500 FYT per kg of feed); 4—0.45% of available P + encapsulated OPP (500 FYT per kg of feed); 5—0.45% of available P + Ladozym proxy from *A. ficuum* (4500 FYT per kg of feed); 6—0.35% of available P + Ladozym proxy from *A. ficuum* (4500 FYT per kg of feed). FEKORD is a nutritional fodder ferment additive; OPP is intracellular phytase from *Obesumbacterium proteus* encapsulated in the *Y. lipolytica Po1f pUV3-Op* yeast.

**Table 3:** The composition of the basal diet for the 6-week- to 8-week-old quails (%) (period 3).

1 kg of the Feed Contains (%)	Groups					
	Group 1	Group 2	Group 3 (5 g/kg)	Group 4 (5 g/kg)	Group 5 (3 g/10 kg)	Group 6 (9 g/10 kg)
Soybean meal	19.95	19.84	20.05	20.16	19.96	19.87
Wheat	31.92	32.49	31.39	30.84	31.86	32.29
Corn	15.00	15.00	15.00	15.00	15.00	15.00
Sunflower seed cake	8.57	8.53	8.60	8.64	8.57	8.54
Full-fat soy	12.00	12.00	12.00	12.00	12.00	12.00
Sunflower oil	3.78	3.63	3.93	4.08	3.80	3.69
OPP	-	-	0.50	0.50	-	-
Ladozym proxy	-	-	-	-	0.03	0.09
Monocalcium phosphate	1.48	0.96	0.97	1.48	1.48	0.97
Limestone Са	6.27	6.53	6.53	6.27	6.27	6.53
Premix	0.50	0.50	0.50	0.50	0.50	0.50
Salt	0.35	0.35	0.35	0.35	0.35	0.35
Methionine	0.09	0.08	0.09	0.09	0.09	0.08
Choline chloride	0.08	0.08	0.08	0.08	0.08	0.08
FEKORD enzyme	0.01	0.01	0.01	0.01	0.01	0.01
Total	100	100	100	100	100	100
100 g of the feed contains (%)						
Metabolizable energy (кcal)	2850	2850	2850	2850	2850	2850
Crude fiber	21.00	21.00	21.00	21.00	21.00	21.00
Raw fiber	5.00	5.00	5.00	5.00	5.00	5.00
Calcium	2.80	2.80	2.80	2.80	2.80	2.80
Phosphorus total	0.79	0.67	0.67	0.79	0.79	0.67
Phosphorus assimilated	0.45	0.35	0.35	0.45	0.45	0.35
Sodium	0.16	0.16	0.16	0.16	0.16	0.16
Chloride	0.26	0.26	0.26	0.26	0.26	0.26
Methionine assimilated	0.38	0.38	0.38	0.38	0.38	0.38

Footnotes: 1—0.45% of available P without any additives; 2—0.35% of available P without any additives; 3—0.35% of available P + encapsulated OPP (500 FYT per kg of feed); 4—0.45% of available P + encapsulated OPP (500 FYT per kg of feed); 5—0.45% of available P + Ladozym proxy from *A. ficuum* (4500 FYT per kg of feed); 6—0.35% of available P + Ladozym proxy from *A. ficuum* (4500 FYT per kg of feed). FEKORD is a nutritional fodder ferment additive; OPP is intracellular phytase from *Obesumbacterium proteus* encapsulated in the *Y. lipolytica Po1f pUV3-Op* yeast.

**Table 4:** The dynamics of the body weight of the quails in the experimental groups (*n* = 10 in each group).

Day of the Quail’s Life	Average Body Weight (g)					
	1 (Positive Control)	2 (Negative Control)	3	4	5	6
BW 7d	39.2 ± 0.52	37.5 ± 0.68	38.2 ± 0.50	39.5 ± 0.55	38.7 ± 0.51	37.88 ± 0.68
Ratio with the positive control	0.00	−1.70	−1.00	+0.30	−0.50	−1.32
Ratio with the positive control (%)	0.00%	−4.34%	−2.55%	+0.77%	−1.28%	3.37%
BW 14d	80.4 ± 1.08	78.7 ± 1.08	78.8 ± 1.06	81.9 ± 0.89	80.9 ± 1.14	79.6 ± 1.38
Ratio with the positive control	0.00	−1.70	−1.60	+1.50	+0.50	−0.80
Ratio with the positive control (%)	0.00%	−2.10%	−1.99%	+1.87%	+0.66%	−1.00%
BW 21d	140.9 ± 1.81	138.9 ± 1.87	139.7 ± 1.47	142.6 ± 1.43	142 ± 1.80	136.3 ± 1.94
Ratio with the positive control	0.0	−2.00	−1.20	+1.70	+1.10	−3.70
Ratio with the positive control (%)	0.0%	−1.42%	−0.85%	+1.21%	+0.78%	−3.26%
BW 28d	169 ± 1.88 ^a^	168.2 ± 2.24	167.9 ± 1.69 ^b^	171.4 ± 1.80 ^a,b^	170.65 ± 2.19 ^a^	164.4 ± 2.18
Ratio with the positive control	0.0	−0.8	−1.10	+2.40	+1.65	−4.6
Ratio with the positive control (%)	0.0%	−0.47%	−0.65%	+1.42%	+0.06%	−2.74%
BW 35d	196.9 ± 3.52 ^a^	195.9 ± 4.90	196.5 ± 2.73 ^a,b^	201.1 ± 3.28 ^a,b^	196.2 ± 2.92 ^a^	192.1 ± 3.61
Ratio with the positive control	0.0	−1.00	−0.40	+4.20	−0.70	−4.80
Ratio with the positive control (%)	0.0%	−0.51%	−0.20%	+2.13%	−0.36%	−2.44%
BW 42d	hens					
	218.5 ± 4.04 ^a^	216 ± 5.39	218.1 ± 3.60 ^a,b^	240.4 ± 4.15 ^a,b^	233.9 ± 6.96 ^a^	215.25 ± 6.09
Ratio with the positive control	0.0	−2.5	−0.40	+21.9	+15.40	−3.25
Ratio with the positive control (%)	0.0%	−1.14%	−1.18%	+10.02%	+7.05%	−1.49%
BW 42d	roosters					
	194.7 ± 4.73 ^a^	190.6 ± 4.89	191.7 ± 4.14 ^a,b^	196.9 ± 3.94 ^a,b^	189.0 ± 4.23 ^a^	180.3 ± 3.18
Ratio with the positive control	0.0	−4.10	−3.00	+2.20	−5.7	−14.40
Ratio with the positive control (%)	0.0%	−2.11%	−1.54%	+1.13%	−2.93%	−7.40%

Footnotes: Groups: 1—0.45% of available P without any additives; 2—0.35% of available P without any additives; 3—0.35% of available P + encapsulated *OPP* (500 FYT per kg of feed); 4—0.45% of available P + encapsulated *OPP* (500 FYT per kg of feed); 5—0.45% of available P + Ladozym proxy from *A. ficuum* (4500 FYT per kg of feed); 6—0.35% of available P + Ladozym proxy from *A. ficuum* (4500 FYT per kg of feed). Statistically significant differences between positive control and negative control after Duncan test were labeled as a and b, respectively (*p* < 0.05). Unlabeled data were not statistically different (Figures S1 and S2).

**Table 5:** Zootechnic features and FCR * of the 42-day-old quails; *n* = 25 in each group.

	Group Number					
	1	2	3	4	5	6
Average daily gain (g)	4.99 ± 0.13 ^a^	4.94 ± 0.15 ^a^	4.91 ± 0.09 ^b^	5.00 ± 0.1 ^b^	4.84 ± 0.11 ^b^	4.73 ± 0.13 ^d^
Feed consumption per head per day (g)	19.66 ± 0.8 ^a^	19.71 ± 0.7 ^b^	19.43 ± 0.8 ^a^	19.59 ± 0.9 ^c^	19.66 ± 0.8 ^a^	19.33 ± 0.9 ^d^
Feed consumption per kg of live weight (kg)	3.75 ± 0.3 ^a^	3.80 ± 0.2 ^c^	3.77 ± 0.4 ^b^	3.73 ± 0.3 ^a^	3.87 ± 0.2 ^d^	3.89 ± 0.1 ^d^
FCR *	3.99	3.99	3.95	3.91	4.06	4.08

* K = k (feed)/M is the formula of the FCR, where k (feed) is the amount of feed consumed by one bird in kg throughout the whole experiment (42 days), and M is the body weight gain. a—*p* < 0.04; b—*p* < 0.01; c—*p* < 0.02; d—did not differ. Footnotes: Groups: 1—0.45% of available P without any additives; 2—0.35% of available P without any additives; 3—0.35% of available P + encapsulated *OPP* (500 FYT per kg of feed); 4—0.45% of available P + encapsulated *OPP* (500 FYT per kg of feed); 5—0.45% of available P + Ladozym proxy from *A. ficuum* (4500 FYT per kg of feed); 6—0.35% of available P + Ladozym proxy from *A. ficuum* (4500 FYT per kg of feed).

**Table 6:** The yield of the cuts (%); *n* = 6 in each group.

	Group Number					
	1	2	3	4	5	6
Yield of carcass parts: breast thigh + drumstick	39.3 ± 2.4 ^a^ 24.8 ± 3.0 ^b^	38.4 ± 1.9 ^c^ 23.8 ± 1.7 ^c^	37.1 ± 1.8 ^b^ 23.5 ± 2.0 ^c^	40.5 ± 3.1 ^a^ 25.1 ± 2.3 ^c^	36.6 ± 3.1 ^b^ 22.4 ± 1.9 ^d^	35.7 ± 3.2 ^a^ 21.6 ± 1.9 ^c^
Yield of muscle parts: breast (fillet) thigh + drumstick	27.3 ± 1.9 ^a^ 17.0 ± 1.2 ^b^	26.5 ± 1.7 ^b^ 16.9 ± 1.2 ^c^	25.3 ± 1.7 ^a^ 16.2 ± 1.3 ^b^	28.4 ± 1.8 18.1 ± 1.3 ^a^	24.2 ± 1.9 ^a^ 15.5 ± 1.4	23.8 ± 1.8 ^b^ 15.1 ± 1.4

Footnotes: Groups: 1—0.45% of available P without any additives; 2—0.35% of available P without any additives; 3—0.35% of available P + encapsulated OPP (500 FYT per kg of feed); 4—0.45% of available P + encapsulated OPP (500 FYT per kg of feed); 5—0.45% of available P + Ladozym proxy from A. ficuum (4500 FYT per kg of feed); 6—0.35% of available P + Ladozym proxy from *A. ficuum* (4500 FYT per kg of feed). The values in each column with different superscripts are different *(p* ≤ 0.05). Unlabeled data were not statistically different.

**Table 7:** The phosphorus and calcium levels in the quails’ tibiae on the 42nd day of age; *n* = 6 in each group.

%	Groups					
	1	2	3	4	5	6
Calcium	20.25	22.19	21.76	21.87	21.90	21.84
Phosphorus	10.48	10.99	10.85	11.18	11.10	11.04

Footnotes: Groups: 1—0.45% of available P without any additives; 2—0.35% of available P without any additives; 3—0.35% of available P + encapsulated *OPP* (500 FYT per kg of feed); 4—0.45% of available P + encapsulated *OPP* (500 FYT per kg of feed); 5—0.45% of available P + Ladozym proxy from *A. ficuum* (4500 FYT per kg of feed); 6—0.35% of available P + Ladozym proxy from *A. ficuum* (4500 FYT per kg of feed).

**Table 8:** Body weight of the 60-day-old quails (g) (M ± m); *n* = 10 in each group.

Group Number	Hens	Roosters	Average Live Weight
1	273.3 ± 5.78 ^a^	211.5 ± 5.50 ^a^	260.9 ± 9.48 ^a^
2	251.3 ± 6.15 ^a^	208.5 ± 2.50 ^a^	242.7 ± 10.29
3	268.2 ± 6.80	206.5 ± 2.80	255.7 ± 9.92
4	276.3 ± 8.86 ^a^	218.4 ± 3.50 ^a^	264.7 ± 10.40 ^a^
5	257.6 ± 6.34 ^a^	196.5 ± 6.50 ^a^	245.4 ± 9.48 ^a^
6	249.1 ± 4.50 ^a^	200.5 ± 4.50 ^a^	239.4 ± 6.15

Unlabeled data were not statistically different. Footnotes: Groups: 1—0.45% of available P without any additives; 2—0.35% of available P without any additives; 3—0.35% of available P + encapsulated *OPP* (500 FYT per kg of feed); 4—0.45% of available P + encapsulated *OPP* (500 FYT per kg of feed); 5—0.45% of available P + Ladozym proxy from *A. ficuum* (4500 FYT per kg of feed); 6—0.35% of available P + Ladozym proxy from *A. ficuum* (4500 FYT per kg of feed). The values in each column with different superscripts are different (*p* ≤ 0.05). Unlabeled data were not statistically different.

**Table 9:** Zootechnic features of the quails and FCR* from 42 to 60 days; *n* = 10 in each group.

	Group Number					
	1	2	3	4	5	6
Average daily gain (g)	2.4 ± 0.2 ^a^	1.51 ± 0.1 ^b^	2.31 ± 0.1 ^c^	2.59 ± 0.2 ^a^	1.90 ± 0.2 ^a^	1.81 ± 0.2 ^c^
Feed consumption per 1 head per day (g)	33.49 ± 2.3	33.02 ± 2.4 ^b^	31.95 ± 2.4 ^c^	32.82 ± 2.3	33.15 ± 2.4 ^b^	32.75 ± 2.1 ^c^
Feed expenses per average body weight (kg)	2.31 ± 0.1 ^a^	2.45 ± 0.1 ^b^	2.25 ± 0.1 ^c^	2.23 ± 0.2 ^b^	2.43 ± 0.1 ^c^	2.46 ± 0.2 ^a^
Egg productivity (%)	77.1 ± 4.8	73.6 ± 4.6	77.8 ± 4.7	86.8 ± 4.9	75.0 ± 4.5	77.8 ± 4.6
FCR *	2.31	2.45	2.25	2.23	2.43	2.46

* K = k (feed)/M is the formula of the FCR, where k (feed) is the amount of feed consumed in kg by one head for 42 days, and M is the body weight gain. Footnotes: Group: 1—0.45% of available P without any additives; 2—0.35% of available P without any additives; 3—0.35% of available P + encapsulated *OPP* (500 FYT per kg of feed); 4—0.45% of available P + encapsulated *OPP* (500 FYT per kg of feed); 5—0.45% of available P + Ladozym proxy from *A. ficuum* (4500 FYT per kg of feed); 6—0.35% of available P + Ladozym proxy from *A. ficuum* (4500 FYT per kg of feed). The values in each column with different superscripts are different (*p* ≤ 0.05). Unlabeled data were not statistically different.

**Table 10:** Chemical assay of macro- and microelements in the quails’ excreta.

Groups	Macro-Element Amount (% of Dry Weight)				Trace Element Amount (µg/g of Dry Weight)	
	P	Mg	K	Ca	Zn	Cu
14 days of the experiment						
1	1.27	0.38	2.06	1.37	1148	92
3	0.96	0.36	2.05	1.01	1158	113
4	1.30	0.46	2.35	1.21	1071	89
5	1.24	0.39	2.19	1.04	1205	91
28 days of the experiment						
1	1.47	0.44	1.91	1.93	681	55
3	1.32	0.45	2.00	1.66	784	65
4	1.38	0.44	1.90	1.63	723	62
5	1.61	0.47	1.89	1.85	642	48
42 days of the experiment						
1	1.42	0.41	1.85	1.82	539	51
3	1.47	0.43	1.79	1.79	565	49
4	1.41	0.46	1.83	1.81	551	48
5	1.53	0.42	1.81	1.93	558	50

Footnotes: Groups: 1—0.45% of available P without any additives; 3—0.35% of available P + encapsulated *OPP* (500 FYT per kg of feed); 4—0.45% of available P + encapsulated *OPP* (500 FYT per kg of feed); 5—0.45% of available P + Ladozym proxy from *A. ficuum* (4500 FYT per kg of feed); *n* = 4 in each group.

**Table 11:** Chemical assay of macro- and microelements in the feed for the quails (start feed).

Diets for the Groups	Macro-Element Amount (% of Dry Weight)				Trace Element Amount (µg/g of Dry Weight)	
	P	Mg	K	Ca	Zn	Cu
1	0.71	0.23	1.31	1.05	373	41
2	0.61	0.28	1.27	1.37	366	64
3	0.73	0.24	1.40	1.42	448	49
4	0.69	0.21	1.30	0.93	246	30
5	0.73	0.22	1.34	1.16	340	42
6	0.58	0.22	1.39	0.90	320	41

Footnotes: Groups: 1—0.45% of available P without any additives; 2—0.35% of available P without any additives; 3—0.35% of available P + encapsulated *OPP* (500 FYT per kg of feed); 4—0.45% of available P + encapsulated *OPP* (500 FYT per kg of feed); 5—0.45% of available P + Ladozym proxy from *A. ficuum* (4500 FYT per kg of feed); 6—0.35% of available P + Ladozym proxy from *A. ficuum* (4500 FYT per kg of feed). *n* = 4 in each group.

## Data Availability

Data are contained within the article and Supplementary Materials.

## Supplementary Materials

The following are available online at https://www.mdpi.com/article/10.3390/vetsci11020091/s1, Figure S1: ANOVA *p*-values and Duncan test adjusted *p*-values for body weight difference 10 selected birds for three groups at 35 days. Footnotes: Data1_B—group 1; Data1_C—group 4; Data1_D—group 5, Figure S2: ANOVA p-values and Duncan test adjusted p-values for body weight difference 10 selected birds for three groups at 42 days. Footnotes: Data1_B—group 1; Data1_C—group 4; Data1_D—group 5, Figure S3: ANOVA p-values and Duncan test adjusted p-values for carcas yield for 6 birds for six groups at 42 days. Footnotes: Data1_B—group 1; Data1_C—group 2; Data1_D—group 3; Data1_E—group 4; Data1_F—group 5; Data1_G—group 6.
