# Supplementary material for: The Efficacy of Encapsulated Phytase Based on Recombinant Yarrowia lipolytica on Quails’ Zootechnic Features and Phosphorus Assimilation
Source: Vet Sci. 2024 Feb 15;11(2):91. doi: 10.3390/vetsci11020091 (PMC10891838; doi:10.3390/vetsci11020091)
Supplement: Supplementary file 1 [file vetsci-11-00091-s001.zip › vetsci-2727183-supplementary.pdf]

[20.10.2023 12:51 "/Data1" (2460250)]

One-Way ANOVA Summary Statistics

**Dataset**	**N**	**Mean**	**SD**	**SE**
Data1_B	10	204,6	5,25357	1,66132
Data1_C	10	221,2	5,59365	51,76887
Data1_D	10	201,3	5,41705	1,71302

Null Hypothesis: The means of all selected datasets are equal
Alternative Hypothesis: The means of one or more selected datasets are different
ANOVA Sum of Mean

Source	DoF	Squares	Square	F Value	P Value
Model	2	2274,86667	1137,43333	38,67359	1,18555E-8
Error	27	794,100000	29,4111111		

At the 0,05 level, the population means are significantly different.

Means Comparison using Bonferroni Test

Dataset	Mean	Difference between Means	Simultaneous Confidence Intervals Lower Limit	Upper Limit	Significant at 0,05 Level
Data1_B	204,6				
Data1_C	221,2	-16,6	-22,79055	-10,40945	Yes
Data1_D	201,3	3,3	-2,89055	9,49055	No
Data1_C	221,2				
Data1_D	201,3	19,9	13,70945	26,09055	Yes

Means Comparison using Scheffe' Test

Dataset	Mean	Difference between Means	Simultaneous Confidence Intervals Lower Limit	Upper Limit	Significant at 0,05 Level
Data1_B	204,6				
Data1_C	221,2	-16,6	-22,88168	-10,31832	Yes
Data1_D	201,3	3,3	-2,98168	9,58168	No
Data1_C	221,2				
Data1_D	201,3	19,9	13,61832	26,18168	Yes

Means Comparison using Tukey Test

Dataset	Mean	Difference between Means	Simultaneous Confidence Intervals Lower Limit	Upper Limit	Significant at 0,05 Level
Data1_B	204,6				
Data1_C	221,2	-16,6	-22,6134	-10,5866	Yes
Data1_D	201,3	3,3	-2,7134	9,3134	No
Data1_C	221,2				
Data1_D	201,3	19,9	13,8866	25,9134	Yes

Power Analysis

Alpha	Total Sample Size	Power	
0,05	30	1,00000	(actual)

Duncan test

```
     diff    lwr.ci    upr.ci    pval
iv-i  16.6  11.623635  21.576365 2.4e-07 ***
v-i   -3.3  -8.276365   1.676365  0.1849
v-iv -19.9 -25.128353 -14.671647 1.2e-08 ***

---
Signif. codes:  0 '***' 0.001 '**' 0.01 '*' 0.05 '.' 0.1 ' ' 1
```

Figure S1. ANOVA *p*-values and Duncan test adjusted *p*-values for body weight difference 10 selected birds for three groups at 35 days. **Footnotes:** Data1_B- group 1; Data1_C - group 4; Data1_D - group 5.

[20.10.2023 12:52 "/Data1" (2460250)]

One-Way ANOVA

Summary Statistics

Dataset	N	Mean	SD	SE
Data1_B	10	222,6	4,40202	1,39204
Data1_C	10	238,7	11,69093	3,697
Data1_D	10	216,9	3,90014	1,23333

Null Hypothesis: The means of all selected datasets are equal
Alternative Hypothesis: The means of one or more selected datasets are different

ANOVA

Source	DoF	Sum of Squares	Mean Square	F Value	P Value
Model	2	2556,46667	1278,23333	22,39023	1,85066E-6
Error	27	1541,40000	57,0888889		

At the 0,05 level,
the population means are significantly different.

Means Comparison using Bonferroni Test

Dataset	Mean	Difference between Means	Simultaneous Confidence Intervals Lower Limit	Upper Limit	Significant at 0,05 Level
Data1_B	222,6				
Data1_C	238,7	-16,1	-24,72481	-7,47519	Yes
Data1_D	216,9	5,7	-2,92481	14,32481	No
Data1_C	238,7				
Data1_D	216,9	21,8	13,17519	30,42481	Yes

Means Comparison using Scheffe' Test

Dataset	Mean	Difference between Means	Simultaneous Confidence Intervals Lower Limit	Upper Limit	Significant at 0,05 Level
Data1_B	222,6				
Data1_C	238,7	-16,1	-24,85177	-7,34823	Yes
Data1_D	216,9	5,7	-3,05177	14,45177	No

Data1_C	238,7				
Data1_D	216,9	21,8	13,04823	30,55177	Yes

Means Comparison using Tukey Test

Dataset	Mean	Difference between Means	Simultaneous Confidence Intervals Lower Limit	Upper Limit	Significant at 0,05 Level
Data1_B	222,6				
Data1_C	238,7	-16,1	-24,478	-7,722	Yes
Data1_D	216,9	5,7	-2,678	14,078	No
Data1_C	238,7				
Data1_D	216,9	21,8	13,422	30,178	Yes

Power Analysis

Alpha	Total Sample Size	Power	
0,05	30	0,99997	(actual)

Duncan test

Posthoc multiple comparisons of means : Duncan's new multiple range test
95% family-wise confidence level

```
$`df$X`
      diff    lwr.ci      upr.ci      pval
iv-i  16.1   9.166823  23.033177   5.7e-05 ***
v-i   -5.7 -12.633177   1.233177   0.1031
v-iv -21.8 -29.084253 -14.515747   9.5e-07 ***

Signif. codes:  0 '***' 0.001 '**' 0.01 '*' 0.05 '.' 0.1 ' ' 1
```

Figure S2. ANOVA p-values and Duncan test adjusted p-values for body weight difference 10 selected birds for three groups at 42 days. Footnotes: Data1_B- group 1; Data1_C - group 4; Data1_D - group 5.

[15.10.2023 15:56 "/Data1" (2460249)]

One-Way ANOVA

Summary Statistics

Dataset	N	Mean	SD	SE
Data1_B	6	137,31667	1,89253	0,77262
Data1_C	6	135,2	0,96954	0,39581
Data1_D	6	133,05	1,56429	0,63862
Data1_E	6	138,95	0,76354	0,31172
Data1_F	6	129,8	1,34759	0,55015
Data1_G	6	126,55	0,60249	0,24597

Null Hypothesis: The means of all selected datasets are equal
Alternative Hypothesis: The means of one or more selected datasets are different

ANOVA

Source	DoF	Sum of Squares	Mean Square	F Value	P Value
Model	5	656,108889	131,221778	80,91230	2,22045E-16
Error	30	48,6533333	1,62177778		

At the 0,05 level,
the population means are significantly different.

Means Comparison using Bonferroni Test

Dataset	Mean	Difference between Means	Simultaneous Confidence Intervals Lower Limit	Upper Limit	Significant at 0,05 Level
Data1_B	137,31667				
Data1_C	135,2	2,11667	-0,2279	4,46124	No
Data1_D	133,05	4,26667	1,9221	6,61124	Yes
Data1_E	138,95	-1,63333	-3,9779	0,71124	No
Data1_F	129,8	7,51667	5,1721	9,86124	Yes
Data1_G	126,55	10,76667	8,4221	13,11124	Yes
Data1_C	135,2				
Data1_D	133,05	2,15	-0,19457	4,49457	No
Data1_E	138,95	-3,75	-6,09457	-1,40543	Yes
Data1_F	129,8	5,4	3,05543	7,74457	Yes
Data1_G	126,55	8,65	6,30543	10,99457	Yes
Data1_D	133,05				

Data1_E	138,95	-5,9	-8,24457	-3,55543	Yes
Data1_F	129,8	3,25	0,90543	5,59457	Yes
Data1_G	126,55	6,5	4,15543	8,84457	Yes

Data1_E	138,95				
Data1_F	129,8	9,15	6,80543	11,49457	Yes
Data1_G	126,55	12,4	10,05543	14,74457	Yes

Data1_F	129,8				
Data1_G	126,55	3,25	0,90543	5,59457	Yes

Means Comparison using Scheffe' Test

Dataset	Mean	Difference between Means	Simultaneous Confidence Intervals Lower Limit	Upper Limit	Significant at 0,05 Level
Data1_B	137,31667				
Data1_C	135,2	2,11667	-0,50022	4,73355	No
Data1_D	133,05	4,26667	1,64978	6,88355	Yes
Data1_E	138,95	-1,63333	-4,25022	0,98355	No
Data1_F	129,8	7,51667	4,89978	10,13355	Yes
Data1_G	126,55	10,76667	8,14978	13,38355	Yes

Data1_C	135,2				
Data1_D	133,05	2,15	-0,46689	4,76689	No
Data1_E	138,95	-3,75	-6,36689	-1,13311	Yes
Data1_F	129,8	5,4	2,78311	8,01689	Yes
Data1_G	126,55	8,65	6,03311	11,26689	Yes

Data1_D	133,05				
Data1_E	138,95	-5,9	-8,51689	-3,28311	Yes
Data1_F	129,8	3,25	0,63311	5,86689	Yes
Data1_G	126,55	6,5	3,88311	9,11689	Yes

Data1_E	138,95				
Data1_F	129,8	9,15	6,53311	11,76689	Yes
Data1_G	126,55	12,4	9,78311	15,01689	Yes

Data1_F	129,8				

Data1_G	126,55	3,25	0,63311	5,86689	Yes

Means Comparison using Tukey Test

Dataset	Mean	Difference between Means	Simultaneous Confidence Intervals Lower Limit	Upper Limit	Significant at 0,05 Level
Data1_B	137,31667				
Data1_C	135,2	2,11667	-0,11967	4,353	No
Data1_D	133,05	4,26667	2,03033	6,503	Yes
Data1_E	138,95	-1,63333	-3,86967	0,603	No
Data1_F	129,8	7,51667	5,28033	9,753	Yes
Data1_G	126,55	10,76667	8,53033	13,003	Yes
Data1_C	135,2				
Data1_D	133,05	2,15	-0,08634	4,38634	No
Data1_E	138,95	-3,75	-5,98634	-1,51366	Yes
Data1_F	129,8	5,4	3,16366	7,63634	Yes
Data1_G	126,55	8,65	6,41366	10,88634	Yes
Data1_D	133,05				
Data1_E	138,95	-5,9	-8,13634	-3,66366	Yes
Data1_F	129,8	3,25	1,01366	5,48634	Yes
Data1_G	126,55	6,5	4,26366	8,73634	Yes
Data1_E	138,95				
Data1_F	129,8	9,15	6,91366	11,38634	Yes
Data1_G	126,55	12,4	10,16366	14,63634	Yes
Data1_F	129,8				
Data1_G	126,55	3,25	1,01366	5,48634	Yes

Power Analysis

Alpha	Total Sample Size	Power	
0,05	36	1,00000	(actual)

```
Posthoc multiple comparisons of means: Duncan's new multiple range test
    95% family-wise confidence level

$`df1$X`
             diff       lwr.ci       upr.ci    pval
```

```
ii-i     -2.116667  -3.6182475  -0.6150858 0.00729 **
iii-i    -4.266667  -5.8446742  -2.6886592 3.5e-06 ***
iv-i      1.633333   0.1317525   3.1349142 0.03401 *
v-i      -7.516667  -9.1442175  -5.8891158 5.4e-11 ***
vi-i    -10.766667 -12.4295804  -9.1037529 2.0e-14 ***
iii-ii   -2.150000  -3.6515809  -0.6484191 0.00652 **
iv-ii     3.750000   2.1719925   5.3280075 2.6e-05 ***
v-ii     -5.400000  -6.9780075  -3.8219925 5.2e-08 ***
vi-ii    -8.650000 -10.2775508  -7.0224492 1.8e-12 ***
iv-iii    5.900000   4.2724492   7.5275508 1.2e-08 ***
v-iii    -3.250000  -4.7515809  -1.7484191 0.00012 ***
vi-iii   -6.500000  -8.0780075  -4.9219925 1.1e-09 ***
v-iv     -9.150000 -10.8129137  -7.4870863 5.6e-13 ***
vi-iv   -12.400000 -14.0896127 -10.7103873 9.4e-15 ***
vi-v     -3.250000  -4.7515809  -1.7484191 0.00012 ***

---
Signif. codes:  0 '***' 0.001 '**' 0.01 '*' 0.05 '.' 0.1 ' ' 1
```

Figure S3. ANOVA p-values and Duncan test adjusted p-values for carcas yield for 6 birds for six groups at 42 days. Footnotes: Data1_B- group 1; Data1_C - group 2; Data1_D - group 3; Data1_E - group 4; Data1_F - group 5; Data1_G - group 6.
